# Malignant Ossifying Fibromyxoid Tumor With Lung Metastasis: A Case Report and Literature Review

**DOI:** 10.7759/cureus.79596

**Published:** 2025-02-24

**Authors:** Kaya Ijiri, Sho Ogata, Kosuke Miyai, Ayano Matsunaga, Chikako Sato, Michiro Susa, Masanori Hisaoka, Susumu Matsukuma

**Affiliations:** 1 Department of Laboratory Medicine, National Defense Medical College Hospital, Tokorozawa, JPN; 2 Department of Pathology and Laboratory Medicine, National Defense Medical College Hospital, Tokorozawa, JPN; 3 Department of Pathology, Japan Self Defense Forces Central Hospital, Setagaya-ku, JPN; 4 Department of Orthopedic Surgery, National Defense Medical College Hospital, Tokorozawa, JPN; 5 Department of Pathology and Oncology, School of Medicine, University of Occupational and Environmental Health, Kitakyushu, JPN; 6 Department of Pathology and Laboratory Medicine, National Defense Medical College, Tokorozawa, JPN

**Keywords:** fluorescence in situ hybridization (fish), immunohistochemistry (ihc), lung metastasis, myoepithelial tumor, ossifying fibromyxoid tumor

## Abstract

Ossifying fibromyxoid tumor (OFMT) is a rare mesenchymal neoplasm of uncertain differentiation, typically exhibiting shell-like ossification and an indolent clinical course. However, some cases demonstrate aggressive behavior with local recurrence or metastasis. A 48-year-old Japanese man presented with a painless right thigh mass. He had been aware of it for 20 years and it had been enlarging slowly for the past year. Pathologic examination revealed that the removed 55-mm tumor chiefly consisted of spindle tumor cells proliferating, in the central area in a hypocellular fashion with scattered ossifications, and peripherally in a more cellular, plexiform-like perivascular fashion with nuclear pleomorphism and frequent mitosis (10 per 50 high-power fields). Tumor cells were positive for keratin, S-100 protein, α-smooth muscle actin, and MUC4, and the Ki67 labeling index was about 40%. S-100 protein immunoreactivity was decreased in the peripheral hypercellular areas. Two months after the surgery, a solitary lung metastasis was evident and was confirmed histologically. Additional fluorescence in situ hybridization examination of the primary tumor cells demonstrated *PHF-1* rearrangement. We concluded that the present case is a rare malignant OFMT. The presence of dense perivascular proliferation and vascular permeation were considered histological indicators for lung metastasis in this case.

## Introduction

Ossifying fibromyxoid tumor (OFMT), a rare mesenchymal neoplasm of uncertain differentiation was first described by Enzinger in 1989 [1-5]. The largest dimension of OFMT usually ranges from 3 to 5 cm but rarely exceeds 15 cm [4]. OFMT is histologically composed of uniform round, ovoid, and spindle tumor cells proliferating in a cord and/or nested fashion and/or a randomly distributed manner within a fibromyxoid stroma. OFMT is usually characterized by a complete or incomplete peripheral bone shell [1,3,4], and this is detectable on computed tomography (CT) examination in at least 60%-70% of OFMT cases [2]. Most OFMTs follow a benign indolent clinical course for more than 10 years [3]. However, some OFMT locally recur and rarely metastasize [1,3,4]. Therefore, OFMT is included in the intermediate category in the latest World Health Organization (WHO) classification [1]. Folpe et al. reported that high cellularity, high nuclear grade, increased mitotic activity, and decreased immunoreactivity for S-100 protein of tumor cells are histological risk-factors predicting aggressive behavior [6]. On the other hand, some authors have observed that OFMT showing even histologically typical features may rarely exhibit local recurrence and metastasize [4]. We recently encountered a rare malignant OFMT that had followed an indolent clinical course and developed lung metastasis after tumor resection. Here, we describe the detailed histopathological features of this case.

## Case presentation

**Figure 1:**
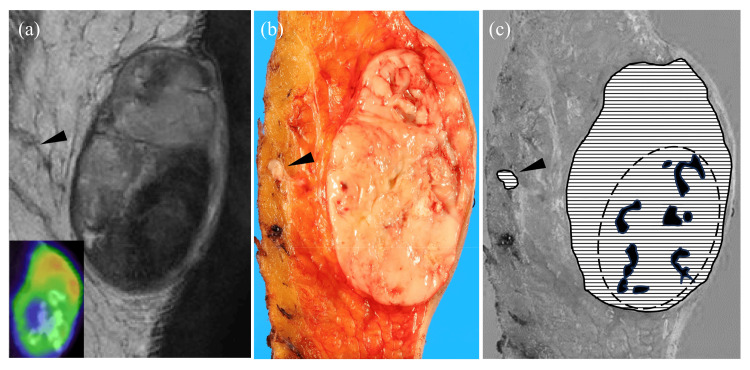
Magnetic resonance imaging (MRI), fluorodeoxyglucose positron emission tomography/computed tomography (FDG-PET/CT), and gross appearance of the removed tumor. (a) A well-circumscribed heterogeneous subcutaneous mass on a T2-weighted image containing low-intensity areas and cord-like projection (arrowhead). FDG-PET/CT revealed FDG uptake in the tumor corresponding to hyperintensity on T2-weighted images (inset). (b) The cut surface of the removed tumor showing a grayish-white appearance with a focal cord-like projection (arrowhead). (c) Schematic illustration of the tumor components (horizontal stripes) and ossifications (black areas). These ossifications were localized roughly in the central area (broken line boundary) of the tumor. An area (indicated by an arrowhead) outside the main tumor showed true vascular invasion by tumor cells.

A 48-year-old Japanese man was admitted with a painless nodule of the right thigh which had been present for 20 years and had slowly enlarged in the previous year. Magnetic resonance imaging (MRI) revealed a well-circumscribed subcutaneous tumor. T2-weighted images mostly exhibited a heterogeneous high intensity with focal low-intensity nodular areas (Figure 1a). Increased vascularity around the tumor was observed after gadolinium contrast administration. Fluorodeoxyglucose positron emission tomography/CT (FDG-PET/CT) showed an FDG-uptake area in the tumor that was concordant with the hyperintensity on the T2-weighted images (Figure 1a, inset) and failed to reveal FDG-uptake in other organs such as lungs. Shortly after the incisional biopsy, the tumor breached the covering skin and caused bleeding, and an extensive resection was performed. Two months after the operation, a solitary nodule in the left lower lobe of the lung was found and this nodule was surgically resected. One year after resection of the main tumor, the patient is alive and well with no local recurrence or distant metastasis.

**Figure 2:**
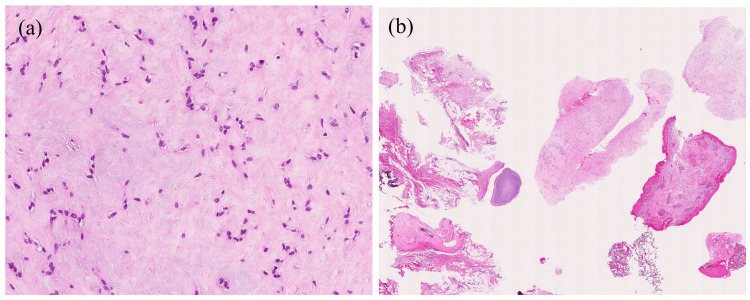
Histopathological findings for the incisional biopsy specimen. (a) In high-power views, the biopsy specimen shows spindle-shaped or ovoid tumor cells with enlarged nuclei within a collagenous stroma. (b) In low-power views, several tissue fragments are observed. There is no evidence of high cellularity or bone formation.

The incisional biopsy specimen consisted of spindle or ovoid tumor cells with swollen nuclei proliferating haphazardly within a collagenous and myxoid stroma (Figure 2a). High cellularity and ossifications were not found (Figure 2b). Some tumor cells were immunoreactive for pan-cytokeratin (AE1/AE3), epithelial membrane antigen (EMA), S-100 protein, or alpha-smooth muscle actin (ASMA), although co-immunoreactivity of all these antigens was not observed. These findings indicated a possible diagnosis of myoepithelial tumor.

**Figure 3:**
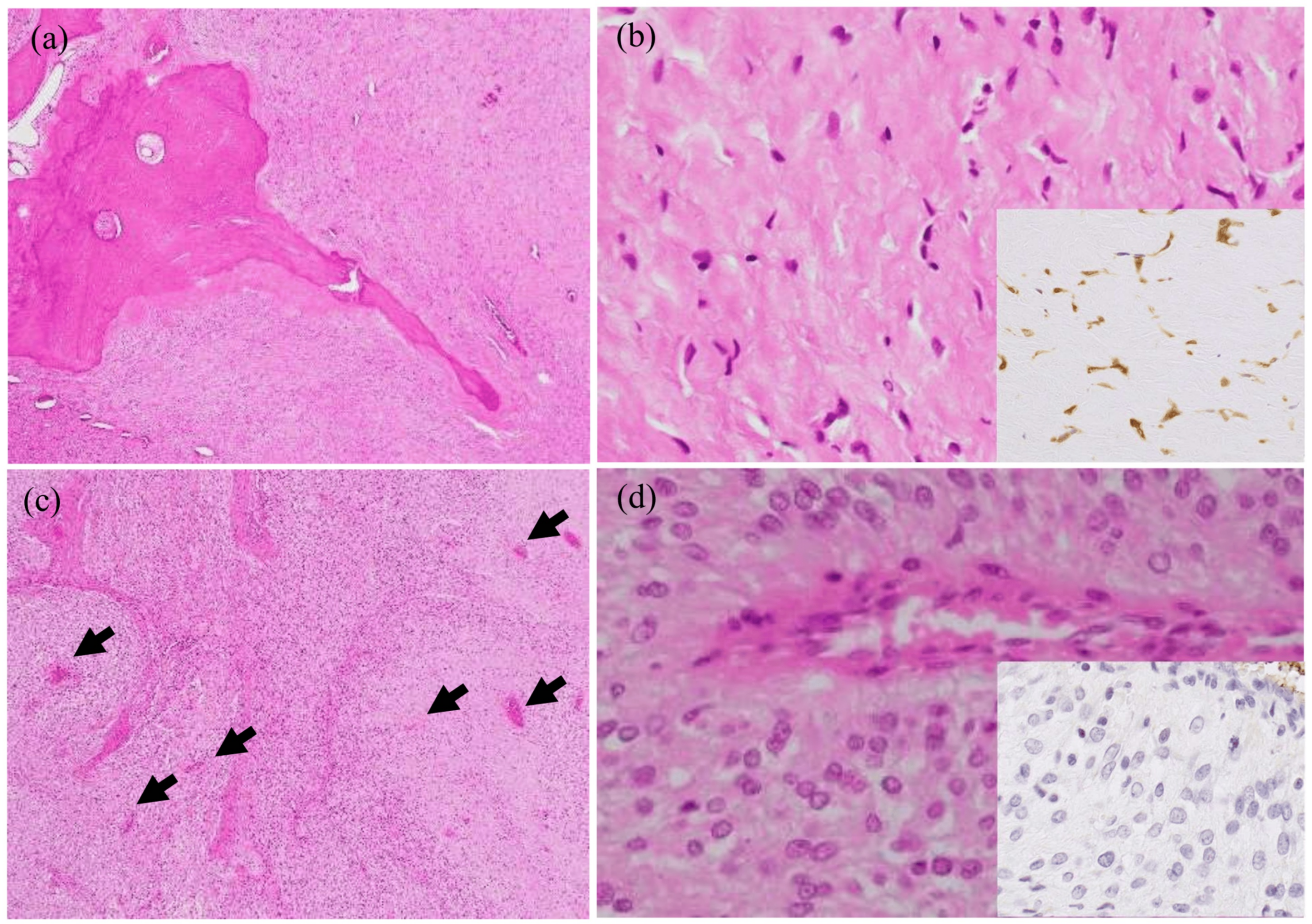
Histopathological findings for the tumor removed from the thigh. Hypocellular tumor cell proliferation concomitant with ossification and myxohyaline stroma in the central area of the tumor (a), and their high-power views (b) showing scattered short spindle tumor cells with immunoreactivity for S-100 protein (inset). In the peripheral areas, plexiform-like fascicular proliferation around blood vessels (arrows)(c), and their high-power views (d) showing more cellular spindle tumor cells with swollen nuclei but with no immunoreactivity for S-100 protein (inset).

**Figure 4:**
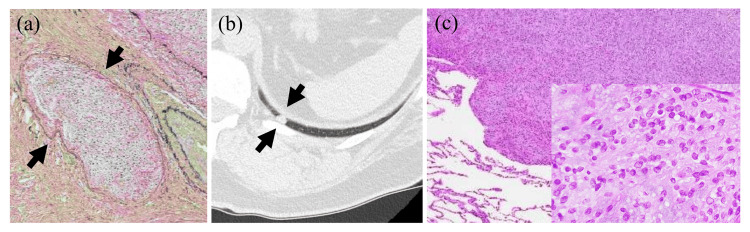
Vascular invasion in the main tumor, and a follow-up chest CT image and histopathological image of the lung metastasis. (a) Distinctive vascular permeation of tumor cells (arrows) was present. (b) A follow-up chest CT two months after the operation demonstrated a solitary nodule in the left lower lung lobe (arrows). (c) The resected lung metastatic nodule showing histological features resembling those of the thigh tumor, with a high-density proliferation of tumor cells within a myxoid stroma (inset).

The resected specimen showed a 55-mm, well-circumscribed, grayish-white tumor mainly involving the subcutaneous tissues (Figure 1b). Shell-like ossification was not grossly found, but small lamellar ossifications, up to 10 mm in size, were microscopically scattered centrally within the tumor (Figure 3a), possibly corresponding to the low-intensity areas on the T2-weighted image of the MRI (Figures 1a, 1c). The tumor mainly consisted of stellate or short-spindle tumor cells with slightly swollen nuclei proliferating haphazardly within a myxohyaline stroma (Figure 3b). Tumor cells were diffusely positive for S-100 protein in areas around the scattered bony deposits (Figure 3b, inset). On the other hand, tumor cells in peripheral zones tended to have larger nuclei, with or without nucleoli and had proliferated in a more cellular, plexiform-like fascicular fashion (Figures 3c, 3d). S-100 protein was negative chiefly in the cellular areas (Figure 3d, inset). In cellular areas, tumor cells showed frequent mitotic figures (10/50 HPFs). True vascular invasion was detected in peripheral areas of the tumor (Figure 4a). Tumor cells were immunohistochemically positive for pan-cytokeratin, EMA, CD56, SOX10, and MUC4. No tumor cells exhibited immunoreactivity for ASMA, p63, glial fibrillary acid protein, CD31, CD34, ERG, or SSX-SS18. Nuclear expressions of both SMARCB1 and H3K27me3 were retained in all tumor cells. The Ki67 labeling index was about 40%. These findings suggested that this tumor was OFMT with a probable malignant potential.

**Figure 5:**
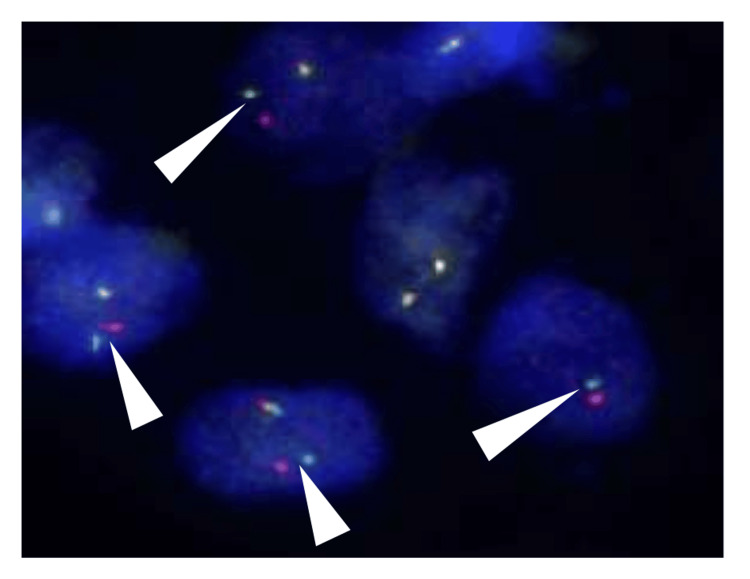
Break-apart fluorescence in situ hybridization (FISH) examination for the PHF-1 gene in the primary thigh tumor. The green signal corresponds to sequences mapping proximal to the *PHF-1* breakpoint region at 6p21.31-p21.32, while the red signal targets sequences distal to the breakpoint at 6p21.32. The separation of green and red signals in 68% (70/102 nuclei, arrowheads) of tumor cells indicates gene rearrangement.

A follow-up CT two months after the operation demonstrated a solitary nodule in the left lower lobe of the lung (Figure 4b). The preoperative PET-CT did not reveal clear evidence of metastasis at the time of evaluation. Removed lung specimens showed a 9 mm-sized solid nodule composed of ovoid cells with swollen nuclei in a myxohyaline stroma (Figure 4c, inset). These features resembled those of the removed OFMT, and it was diagnosed as a lung metastasis of the OFMT. Additional fluorescence in situ hybridization (FISH) for *PHF-1* in the primary tumor, using the ZytoLight SPEC *PHF-*1 dual-color break-apart probe (ZytoVision, Bremerhaven, Germany), demonstrated break-apart signals in 68% of the tumor nuclei (70/102) (Figure 5). These findings strongly supported the diagnosis of malignant OFMT.

## Discussion

The present tumor is considered a rare malignant OFMT showing distinctive lung metastasis. Folpe et al. proposed criteria for predicting malignant clinical courses as follows: tumors with 1) high nuclear grade or 2) high cellularity and mitotic counts >2 mitotic figures/50 HPFs [6]. The present tumor displayed high-grade nuclear atypia with increased mitotic figures and partially high cellularity; thus it fits these criteria and was classified as “malignant” OFMT. Previous reports indicated that 20% to 60% of malignant OFMTs (using Folpe’s criteria) exhibited distant metastases, in which the lungs were the most common site, as in the present case [6-9]. On the other hand, Miettinen et al., who examined 104 OFMT cases with at least focal areas of typical ossification, reported that these tumors did not have any distant metastases. Still, some did show local recurrence [10].

Our review of the literature disclosed eight previous OFMT cases with pulmonary metastasis, and these are summarized in Table 1 [11-18]. The cases ranged in age from 32 to 71 years, with a predominance of males and a tendency for tumors to arise in the extremities (legs, thighs, buttocks) or trunk (back, chest wall, ribs). Histologically, most of these tumors were diagnosed as “atypical” or “malignant.” Vascular invasion, a possibly most useful marker for predicting pulmonary metastasis, was detectable in only two (22%) of the nine pulmonary metastatic cases (including the present case). In our view, the case reported by Minami et al. was similar to the present case because it exhibited vascular invasion and was classified as malignant histologically [11]. A prominent plexiform-like perivascular growth pattern and true vascular invasion characterize the present case. The former type of lesion was not found in our review of previous articles, and we should expect the latter feature to be strongly associated with the subsequent pulmonary metastatic event.

**Table 1:** Comparison of clinicopathological features between the present case and previously reported cases of ossifying fibromyxoid tumor metastatic to the lung.

Author name (ref. #)	Age (years)	Male/female	Primary location	Histological grade	Venous invasion	Perivascular pattern
Lastra et al., 2014 [12]	55	F	Left ankle	Atypical/malignant	No	No
Nishio et al., 2002 [13]	52	M	Right foot	Atypical	No	No
Schaffler et al., 1997 [14]	32	M	Left back	Atypical/malignant	No	No
Sarraj et al., 2007 [15]	71	M	Left leg	Uncertain	No	No
Sbaraglia et al., 2020 [16]	65	F	Rib	Malignant	No	No
Minami et al., 2001 [11]	70	M	Right buttock	Malignant	Yes	No
Kilpatrick et al., 1995 [17]	68	M	Right hip	Typical	No	No
Patel et al., 2023 [18]	64	M	Chest wall	Malignant	No	No
Present case	48	M	Right thigh	Malignant	Yes	Yes

A peripheral bony shell is considered a key histological finding for a diagnosis of OFMT [1-4]. However, in a small biopsy specimen, this feature may be obscure. Indeed, in the present case, the biopsy specimen did not contain osseous lesions. Miettinen et al. reported that no ossification was observed in 16 tumors (15%) out of 104 OFMTs [10]. Folpe et al. found that 63% (44 of 70) of OFMTs exhibited at least focal ossification, and in 89% and 11% of these 44 cases, ossification within the tumor was distributed at the periphery/septum or randomly, respectively [6]. Histological examination of the present tumor revealed small deposits of bone focally and centrally, but not peripherally. In addition, interestingly, S-100 protein immunoreactivity was preserved near the bone-scattered areas but was diminished in the peripheral areas without bone deposition. These findings suggested that malignantly transformed components with attenuated S-100 protein expression may have spread, beyond the pre-existing area of scattered bone in a benign tumor, into the surrounding tissues.

Differential diagnosis of OFMT should include myoepithelioma/myoepithelial carcinoma because both tumors display S-100 protein immunoreactivity [2,9,19]. MUC4 is usually negative in myoepithelial tumors but is positive in 29% (five of 17) of OFMT cases [20]. Additional immunohistochemical markers such as GFAP, which may be positive in myoepithelioma/myoepithelial carcinoma, and SMARCB1, which may show mosaic loss specifically in OFMT [1,2], are useful for such differential diagnosis. The rearrangement of the *PHF-*1 gene was confirmed in the present case. This is specifically found in 50% of OFMTs and is detected at a similar percentage across typical, atypical, and malignant OFMT cases [1]. A combined assessment of proper immunostaining panels and molecular analysis could lead to a correct diagnosis.

## Conclusions

We present a case of rare malignant OFMT metastasizing to the lung, with a previous long-standing indolent clinical course. In this case, the presence of dense perivascular proliferation and vascular permeation were considered to be histological indicators for lung metastasis. It is important to recognize that even indolent OFMT has the potential for distant metastasis, and thus follow-up of a very long duration is recommended unless the tumor has been excised.
